# Controlled delivery and minimally invasive imaging of stem cells in the lung

**DOI:** 10.1038/s41598-017-13280-9

**Published:** 2017-10-12

**Authors:** Jinho Kim, Brandon Guenthart, John D. O’Neill, N. Valerio Dorrello, Matthew Bacchetta, Gordana Vunjak-Novakovic

**Affiliations:** 1 Department of Biomedical Engineering, Columbia University, New York, NY USA; 2 Department of Surgery, Columbia University, New York, NY USA; 3 Department of Pediatrics, Columbia University, New York, NY USA; 4 Department of Medicine, Columbia University, New York, NY USA

## Abstract

Intratracheal delivery of stem cells into injured or diseased lungs can provide a variety of therapeutic and immunomodulatory effects for the treatment of acute lung injury and chronic lung disease. While the efficacy of this approach depends on delivering the proper cell dosage into the target region of the airway, tracking and analysis of the cells have been challenging, largely due to the limited understanding of cell transport and lack of suitable cell monitoring techniques. We report on the transport and deposition of intratracheally delivered stem cells as well as strategies to modulate the number of cells (e.g., dose), topographic distribution, and region-specific delivery in small (rodent) and large (porcine and human) lungs. We also developed minimally invasive imaging techniques for real-time monitoring of intratracheally delivered cells. We propose that this approach can facilitate the implementation of patient-specific cells and lead to enhanced clinical outcomes in the treatment of lung disease with cell-based therapies.

## Introduction

Despite notable advances in biomedical research, drug development, and clinical management, lung disease remains a leading cause of morbidity and mortality^1,2^. At least 210 million people around the world suffer from chronic respiratory conditions, with chronic obstructive pulmonary diseases (COPD) accounting for nearly 3 million deaths a year, making it the third leading cause of death worldwide^3,4^. Additionally, acute lung injury^5,6^, respiratory infections such as pneumonia and influenza^7–9^, and asthma^10,11^ account for millions of additional deaths each year. While lung transplantation is the only definitive option for patients with end-stage lung disease, regenerative medicine and tissue engineering have yet to provide a solution for the critical shortage of donor lungs^12,13^. Therefore, more effective strategies are needed to reduce the global burden of respiratory disease and alleviate the donor lung shortage^14^.

Cell-based therapies have emerged as a promising approach that could impact clinical outcomes for patients with lung disease or acute lung injury. In particular, mesenchymal stem cells (MSCs) have been extensively tested in animal models and clinical trials^15,16^. Through a variety of paracrine actions, MSCs have been shown to induce cell proliferation and angiogenesis, and rescue near-apoptotic cells. MSCs also have the ability to act as antigen-presenting cells, modulate the local immune response, and enhance natural repair mechanisms through activation of endogenous progenitors and mature cells^17^. Accordingly, pre-clinical studies of MSC therapy in acute respiratory distress syndrome (ARDS)^18^, cystic fibrosis^19^, and emphysema^20^ have revealed potential therapeutic benefits of MSCs in the treatment of these diseases.

A number of clinical studies have demonstrated the safety of MSCs for treating lung disease. However, the efficacy of MSCs reported during phase I trials was only marginal^21^. To enhance therapeutic efficacy, parameters such as the number of cells (i.e., cell dose), delivery mechanism, and delivery route (e.g., intravenous vs. intratracheal) need to be optimized for disease- and patient-specific applications^22^. For example, while the cell dosages used in clinical studies displayed good safety profiles with limited efficacy, increasing total cell number may enhance therapeutic outcomes^16^, a dose-response effect that has yet to be fully elucidated. Unlike intravenous cell injection – where most cells are trapped in the pulmonary microvasculature due to their large size and surface-adhesion receptors – administration of MSCs through the trachea via liquid bolus (i.e., intratracheal administration) could increase the chance for the cells to reach targeted lung regions and augment therapeutic effects^23^.

The underlying mechanism of the intratracheal cell delivery strategy is similar to that of surfactant delivery, as both applications involve deposition of therapeutic materials (i.e., cells or surfactant) on the airway surfaces via liquid plugs traveling through the pulmonary airways. Many researchers have investigated fluid mechanics and transport phenomena in surfactant replacement therapy^24–26^. In addition, cell delivery into airways of small and large animal lung have been demonstrated to show the therapeutic efficacy^27,28^. However, current incomplete understanding of transport behaviors and deposition mechanisms associated with cell delivery via the lung airways has largely impeded the establishment of effective strategies for intratracheal cell delivery. Furthermore, cell delivery optimization has been hindered by the lack of effective means to continuously monitor the fate of administered cells (e.g., migration, engraftment, and function) in the lungs^29^.

To enhance the therapeutic efficacy of stem cell-based therapies and lung regeneration, we studied the transport and deposition of MSCs administered intratracheally into the lungs. In addition, we established the minimally invasive optical fiber-based imaging to investigate cell delivery. To facilitate translation, systematic experimental studies were conducted using rat, porcine, and human lungs with cell deposition on the tracheal surface via instillation of micro-volumes of liquid carrying the cells. The cells deposited on the tracheal surface were visually inspected using optical-fiber imaging probes *in situ*. Cell delivery into the distal gas exchange regions (i.e., alveoli) of rat lungs was confirmed using minimally invasive, real-time transpleural imaging.

## Results

### Intratracheal cell delivery and *in situ* visualization

**Figure 1:**
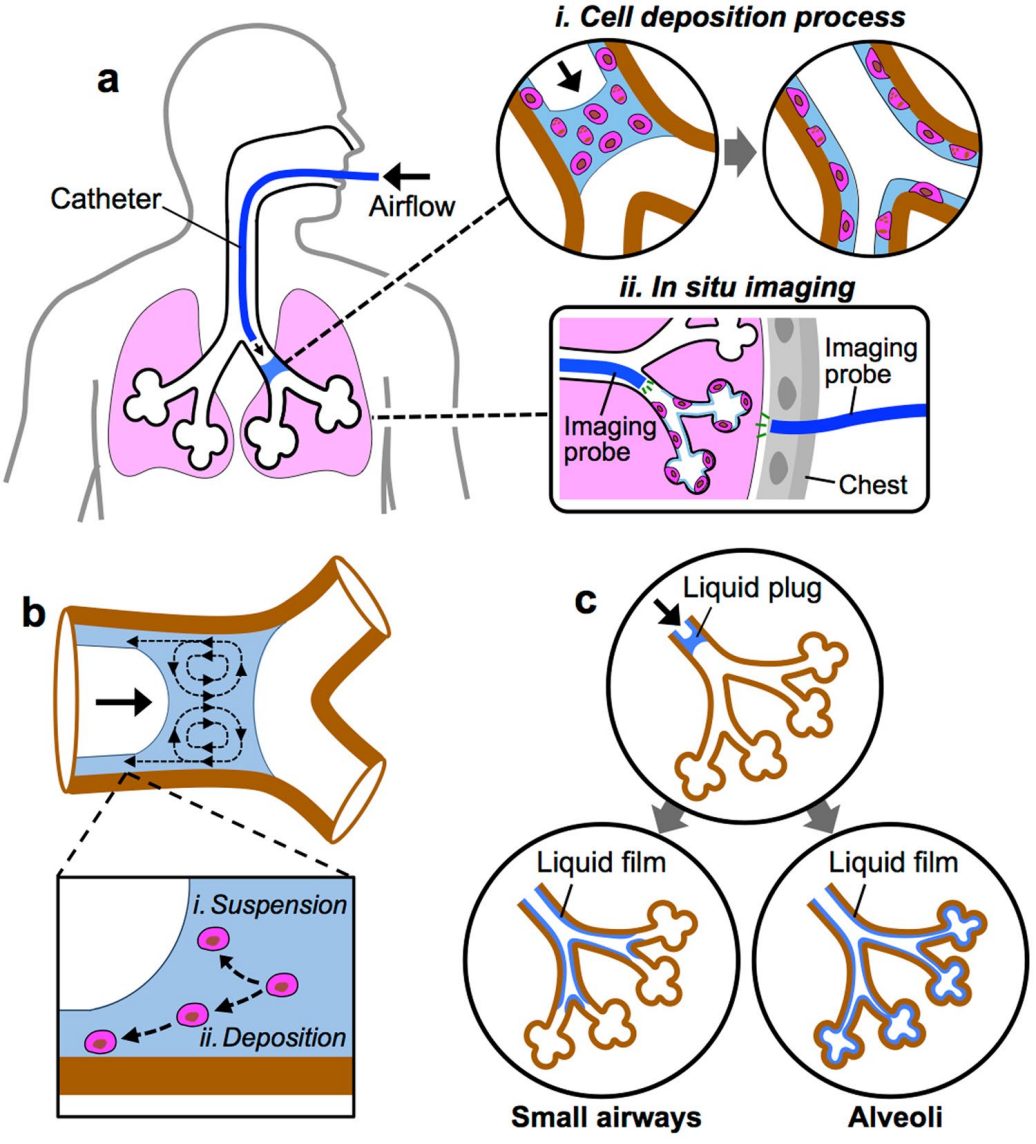
Intratracheal delivery and *in situ* imaging of cells in the lung. (**a**) Deposition of cells onto airway surface by instillation of a small volume of liquid using a catheter. Insets showing (*i*) cell deposition process and (*ii*) *in situ* imaging. (**b**) Typical internal circulatory flow pattern within a liquid plug traveling in the airway that results in (*i*) suspension and (*ii*) deposition of cells. (**c**) Deposition of liquid film in selected lung regions (e.g., small airways or alveoli) by adjusting flow conditions (e.g., plug speed and volume).

Deposition of cells on the airway surfaces was achieved by instillation of small volumes (i.e., microliters to milliliters) of cell suspension through the airway (Fig. 1a). During intratracheal delivery, cells are first deposited on the airway surface via a traveling liquid plug, and then either adhere to or migrate along the surface of the airway. Cell deposition (the first step above) can be mainly due to the capture of cells within the liquid film being deposited at the rear end of the traveling plug. In a clinical setting, a specialized bronchoscope can be used to form a liquid plug in a proximal airway near the target lung region in the diseased or injured airway. Then, the plug is pushed along the airway toward the target region by applying positive air pressure to the liquid plug by ventilating the lung. As the liquid plug travels, a thin film is deposited on the airway surface^30,31^, enabling cell attachment to the airway. The cell delivery process is complete when the decreasing volumes of all liquid plugs are finally exhausted due to film deposition (Fig. 1a, *i*). Cells deposited on the surfaces of proximal and distal airways were visualized *in situ* using imaging probes inserted into the airway or into a small incision made in the chest, respectively (Fig. 1a, *ii*).

The transport behaviors of cells suspended in a liquid plug traveling through airways can be determined by the flow pattern of the plug (Fig. 1b). In fact, an internally circulating flow is generated in a liquid plug in motion^32^ due to the differences between the higher viscous resistance near the airway surface and lower resistance near the center of the plug^33^. As a result, cells near the airway surface at the rear end of the plug will be deposited along the airway surface, while the remaining cells can be well-mixed and stay suspended in the liquid plug by the circulating flow^34^. Uniform cell distributions across airway surfaces would be achieved, as cells could follow these flow patterns. The thickness of the liquid film generated on the airway surface is proportional to the plug capillary number, which is a function of the liquid viscosity, surface tension, and plug speed^24–26^. Because cells are seeded by liquid film generated, cell density at the surfaces (i.e., cell-seeding density) can be manipulated with the plug capillary number. For example, because the thickness of the liquid film is proportional to the traveling speed of the plug^30,31^, a greater number of cells can be trapped and deposited on the airways by a plug traveling faster. Similarly, cell-seeding density can increase with cell concentration within the liquid plug.

The total airway surface area covered with cells (i.e., cell coverage area) can be controlled by varying the flow conditions during instillation (Fig. 1c). For instance, greater cell coverage areas can be achieved by instilling increasing volumes of liquid plugs because larger plugs travel longer distances and thus cover more airway surfaces. In addition, the traveling speed of the plugs influences the thickness of the liquid film deposition. Therefore, fast traveling plugs generate thicker liquid film and thus travel shorter distances, while plugs traveling at slower speeds increase cell coverage areas. As a result, deposition of cells in selected airway regions (e.g., small airways or alveoli) can be achieved by specifying plug instillation flow conditions along with the initial plug volume introduced into the airway.

### *In vitro* studies of cell transport

**Figure 2:**
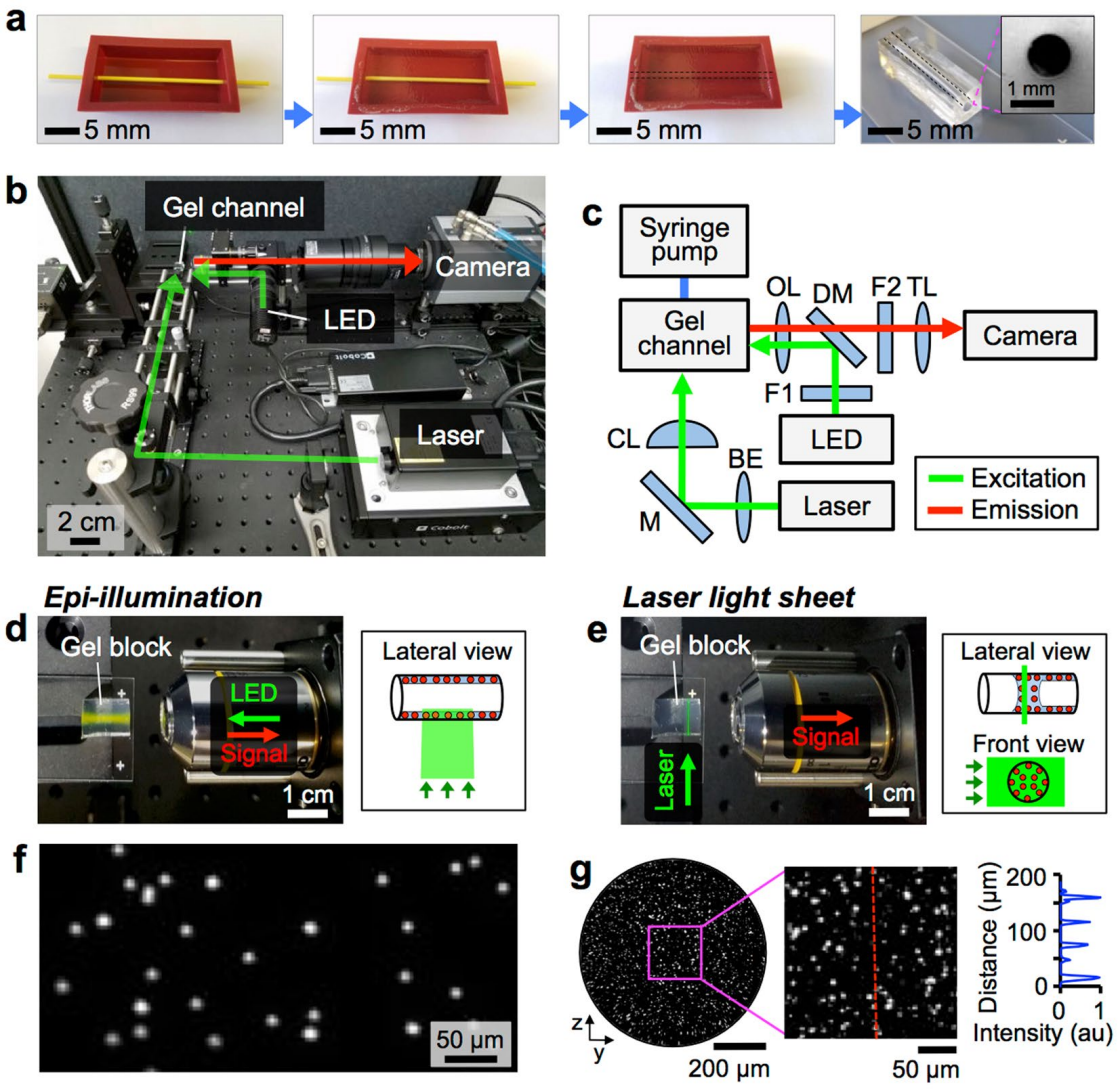
Cell delivery using gelatin tubes. (**a**) Fabrication of gelatin tubes. (**b**) Photograph and (**c**) light path schematic of the imaging setup. BE: beam expander, M: mirror, CL: cylindrical lens, F1 & F2: filters 1 & 2, OL: objective lens, DM: dichroic mirror, TL: tube lens. (**d**) LED-based epi-illumination. (**e**) Laser light sheet illumination. (**f**) A fluorescent image of 10-μm particles deposited on the inner channel surface obtained using LED imaging. (**g**) Imaging 1-μm particles suspended in a liquid plug within a channel using laser-sheet imaging.

We first investigated the transport behaviors of cells *in vitro* using gelatin-based tubular channels (Fig. 2a) prepared by previously established methods for fabricating hydrogel-based microfluidic structures^35^. A custom-built florescent microscope system was used to investigate the transport of MSCs, which were labeled with quantum dots (Qdots) (Supplementary Fig. 1), by a liquid plug traveling within the gelatin tube (Fig. 2b,c). To characterize the hydrodynamics of cell motion, we first used commercially available fluorescently labeled microparticles of varying sizes (diameter: 1 μm and 10 μm). We used our customized imaging system (developed in-house) that employs LED-based epi-illumination and laser-sheet illumination techniques to obtain concurrent views of the channel surfaces and channel cross-section, respectively. While LED imaging proved useful for inspecting and quantifying particles or cells deposited on the inner surface of the gelatin channel (Fig. 2d), laser -sheet imaging (light-sheet thickness: <5 μm) enabled us to study the transport and settlement of individual particles and cells^36^ (Fig. 2e, Supplementary Fig. 2). Using LED imaging, particles (diameter: 1 μm and 10 μm) deposited on the channel surface were visualized clearly following instillation of a liquid plug (Fig. 2f, Supplementary Video 1). Laser-sheet imaging, compared with LED imaging (Supplementary Fig. 3), provided significantly enhanced image quality of particles (diameter: 1 μm) filled in a channel as the light intensity profile measured along a vertical line drawn in the image showed significantly greater contrast (Fig. 2g).

### Hydrodynamics of cell motion in gelatin channels

**Figure 3:**
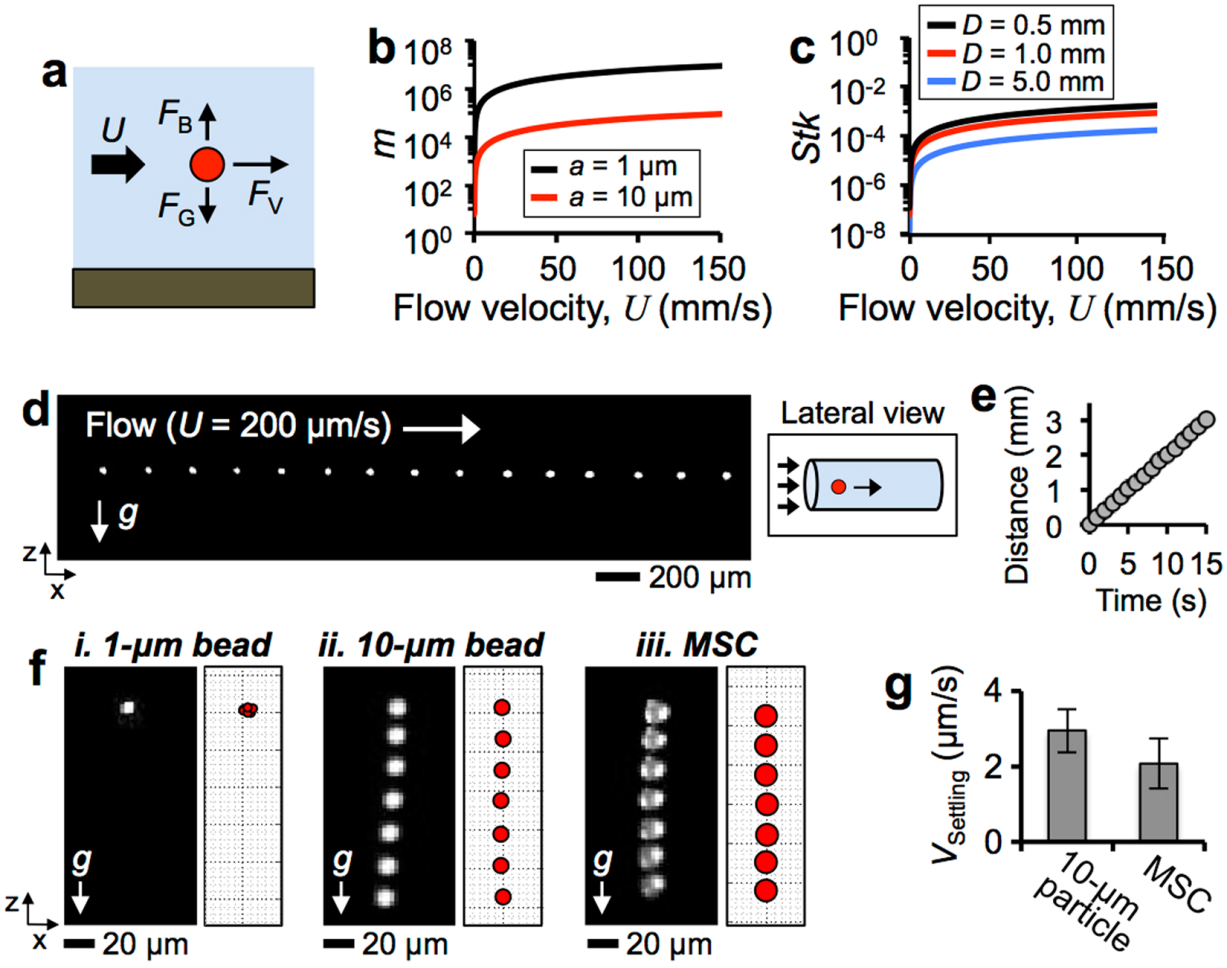
Hydrodynamic behaviors of cells. (**a**) Force diagram of a particle in liquid flow. (**b**) *m* = *F*_V_/(*F*_G_ − *F*_B_) as a function of *U*. (**c**) Particle Stokes number *Stk = U*ρ_P_ *d* ^2^/18 μ*D* of a 10-μm particle for various tube diameters *D* as a function of *U*. (**d**) Trajectory of a MSC carried by flow at *U* = 200 μm/s and (**e**) travel distances measured over 15 s. (**f**) Experimentally obtained (left) and simulated (right) trajectories of (*i*) 1-μm particle, (*ii*) 10-μm particle, and (*iii*) MSC settling without flow. (**g**) Settling velocities of a 10-μm particle and MSC determined experimentally.

When a cell suspended in liquid is in motion, its hydrodynamic behavior is mainly influenced by the viscous force (*F*_V_), gravity (*F*_G_), and buoyancy force (*F*_B_)^34^ (Fig. 3a). Assuming a cell as a spherical particle of diameter *d*, with fluid viscosity μ and velocity *U*, the viscous force is *F*_V_ = 3πμ*dU*. The net effect of gravity becomes *F*_G_ − *F*_B_ = (ρ_P_ − ρ_F_)g*d* ^2^, where ρ_P_ and ρ_F_ are the density of particle and fluid, respectively. The magnitude of the ratio *m* = *F*_V_/(*F*_G_ − *F*_B_) is an index of the relative importance of the viscous force and gravity to the transport of the particle, where *m* > 1 when viscous force dominates and *m* < 1 when gravity dominates. For small particles (e.g., diameter: 1 μm and 15 μm), *m* >> 1 for a wide range of flow velocities (*U* = 0.01–150 mm/s), indicating that the transport behavior of a MSC (diameter: ~15 μm^37^) is mostly influenced by viscous forces within a liquid plug (Fig. 3b). The particle Stokes number *Stk* = *U*ρ_P_ *d* ^2^/18μ*D* can indicate how closely a particle can follow the fluid streamlines in a tube of a diameter *D*. For *Stk* << 1, particles can readily adopt the fluidic motion, and so smaller particles follow the flow better than larger particles. *Stk* calculated for a MSC within channels of different diameters *D* for *U* = 0.01–150 mm/s shows that the cells can travel readily with liquid plug when *Stk* << 1 (Fig. 3c).

Using the laser light sheet imaging technique, we investigated the hydrodynamic behaviors of MSCs traveling in liquid plugs within a gelatin tube (*D* = 1 mm). The trajectory of a MSC within a continuous flow in a plug traveling at a very slow speed (*U* = 200 μm/s, Reynolds number Re = 229) in a gelatin tube was obtained from a video recorded for 15 s during cell transport. The trajectory of the cell was not affected by gravity, as the cell traveled in a direction parallel to the axis of the tube (Fig. 3d). The velocity of the cell, calculated from the slope of the time-dependent travel distance curve, was ~200 μm/s, which was identical to the flow velocity of the plug in the tube (Fig. 3e). For a MSC under these flow conditions, *m* = 9.66 × 10^4^ >> 1 and *Stk* = 1.29 × 10^−6^ << 1 indicating that cells can readily follow the circulating motion of the flow within liquid plugs traveling through the airways due to the viscous force (Fig. 1b).

We also investigated the gravity settling behavior of a MSC, and the 1-μm and 10-μm particles in the absence of flow (Fig. 3f, *i*-*iii*, Supplementary Videos 2–4). Using a pipette, a liquid plug containing particles or MSCs was introduced gently into a gelatin tube, which was initially filled with air, to mimic pulmonary small airways. Experimental data obtained during settlement of the particles or cells were compared with simulations using MATLAB (please see Supplementary Information for details). Settling velocity *V*_Settling_ = ρ_P_g*a* ^2^/18 μ for a 1-μm particle, 10-μm particle, and MSC were approximately 2.45 × 10^−2^ μm/s, 2.45 μm/s, and 2.32 μm/s, respectively. Thus, 10-μm particles and MSCs eventually settled at the bottom of the gelatin tube channel (*D* = 1 mm) within 10 min, while 1-μm beads remained suspended for a long period of time. Multiple 10-μm particles and MSCs (>12 each) were tracked to determine their *V*_Settling_, which were determined to be 2.95 μm/s ± 0.57 μm/s and 2.08 μm/s ± 0.67 μm/s, respectively. These experimental results were consistent with theoretically predicted values, and showed that MSCs settled approximately 1.4 times slower than 10-μm particles (Fig. 3g).

### Deposition of cells in the gelatin channel via liquid plug instillation

**Figure 4:**
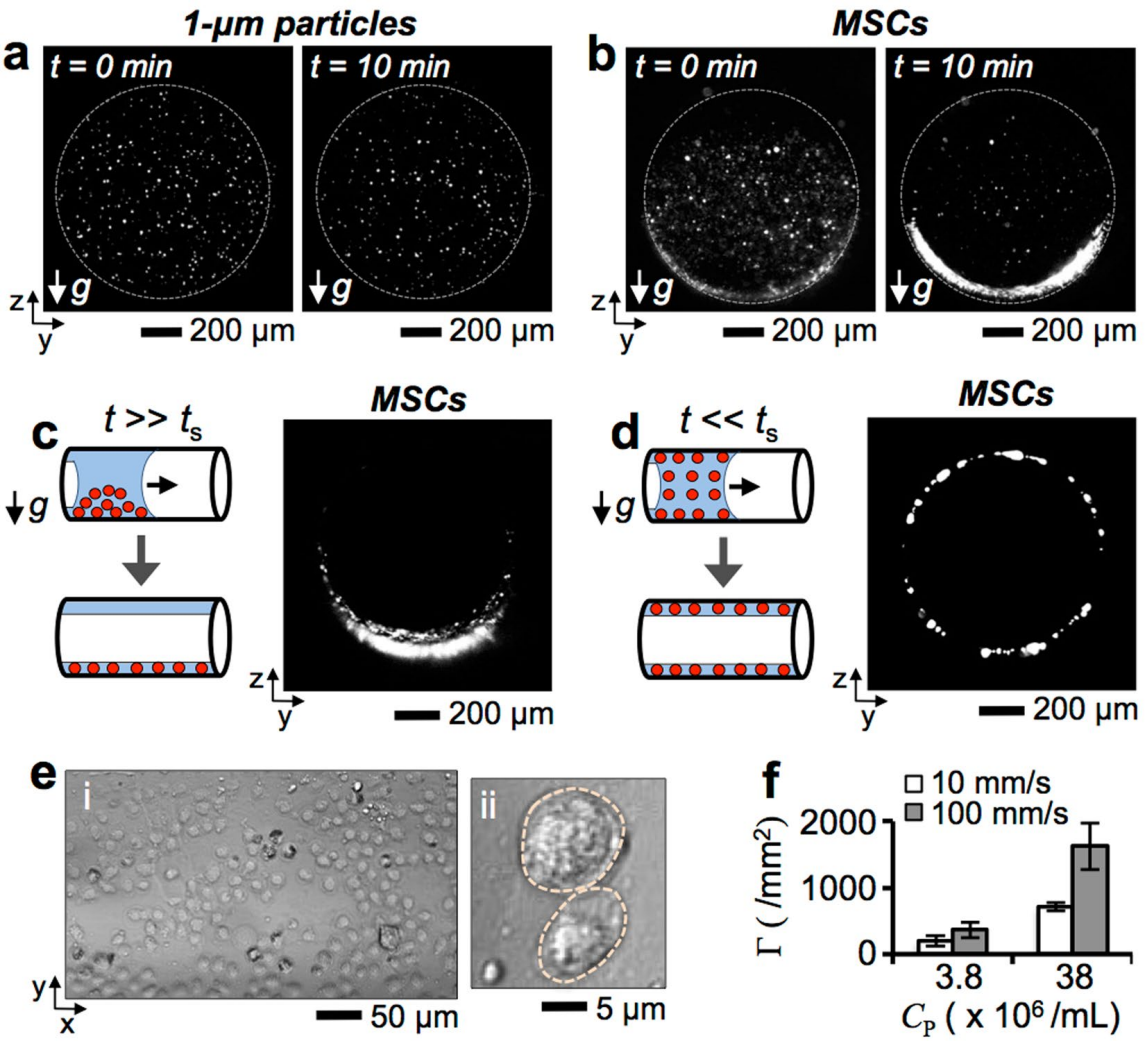
Deposition of MSCs onto the inner surface of a gelatin tube. Cross-sectional images of gelatin tubes filled with (**a**) 1-μm particles and (**b**) MSCs were obtained at *t* = 0 min and 10 min using the laser light sheet microscopy. Cross-sectional images of gelatin tubes deposited with MSCs with (**c**) *t* >> *t*_s_ and (**d**) *t* << *t*_s_, where *t*_s_ ~ 10 min. (**e**) Microscopic images of (*i*) inner surface of a gelatin tube and (*ii*) magnified view of MSC instillation into the tube. (**f**) Surface coverage density Γ of MSCs at various cell concentrations *C*_P_ and flow instillation velocities *U*.

To investigate cell deposition, MSCs were delivered onto the inner surface of the gelatin tube via liquid plug instillation. Using light-sheet imaging, the cross-section of liquid plugs containing 1-μm beads or MSCs, respectively, were visually inspected for 10 min after being introduced into the gelatin tubes (*D* = 1 mm). We defined the settling time *t*_s_, as the time that takes for a particle or cell to travel the distance equivalent to the tube diameter (*D* = 1 mm) by gravity settling at its settling velocity *V*_Settling_. For a 1-μm particle and MSC, *t*_s_ are approximately 680 min and 8 min, respectively. Experimental results showed that while 1-μm particles were continually suspended in liquid, the majority of MSCs were deposited on the channel bottom surface within 10 min (Fig. 4a,b, Supplementary Fig. 4). Specifically, when a liquid plug carrying MSCs was instilled into the gelatin channel after 10 min of plug introduction (i.e., *t* >> *t*_s_, where *t* is the plug instillation time), most of the cells were deposited at the bottom of the channel by gravity (Fig. 4c). In contrast, when a plug was instilled just after the introduction into the channel (i.e., *t* << *t*_s_), cells were deposited uniformly across the channel surface (Fig. 4d). Thus, to achieve uniform cell distribution on airway surfaces, it is critical to minimize the settlement of the cells.

Attachment of MSCs onto the gelatin channel surface was inspected at cell concentrations of *C*_P_ = 3.8 × 10^6^ cell/mL and 3.8 × 10^7^ cell/mL, and plug velocities *U* = 10 mm/s and 100 mm/s. For uniform cell deposition, liquid plugs were instilled quickly following introduction into the channel (i.e., *t* << *t*_s_). The surface coverage density (or seeding density) Γ of the cells was determined from microscopic images obtained under the epi-illumination (Fig. 4e, *i*-*ii*). Results show that the surface coverage density Γ increased with cell concentration *C*_P_. In addition, higher densities were achieved with greater *U*. This proportional relationship reflects the finding that a faster liquid plug leaves a thicker liquid film behind^38^ because cells at the plug’s rear end had increased chance for deposition on the surface (Fig. 4f).

### Cell delivery into the rat trachea and *in situ* imaging

**Figure 5:**
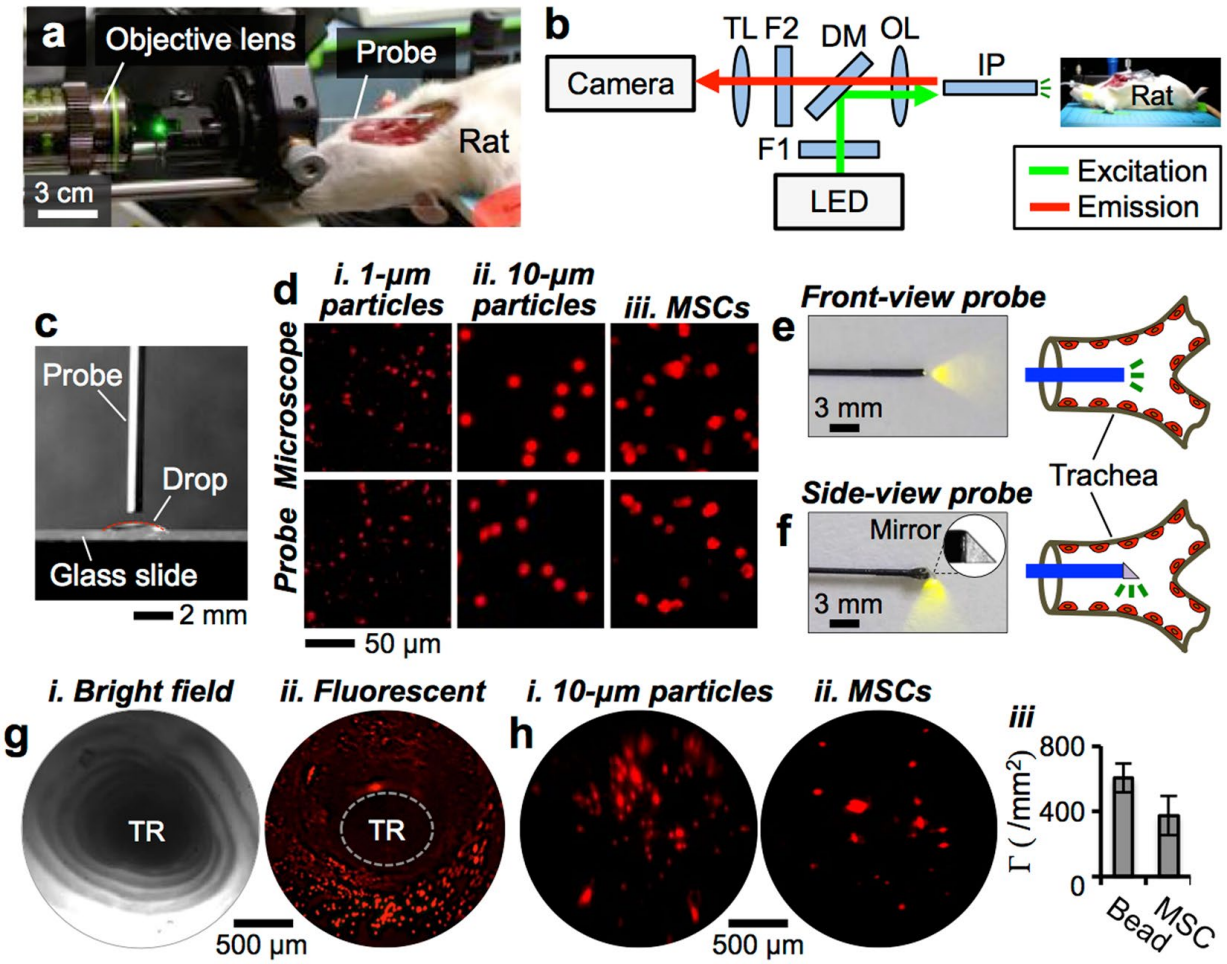
MSC delivery into the rat trachea and *in situ* visualization. (**a**) Photograph of the imaging setup for visualizing the rat trachea and (**b**) light-path diagram of the imaging setup. IP: imaging probe, OL: objective lens, DM: dichroic mirror, F1 & F2: optical filters 1 & 2, TL: tube lens. (**c**) Imaging probe characterization to visualize (**d**) (*i*) 1-μm, (*ii*) 10-μm particles, and (*iii*) MSCs suspended in liquid droplets and placed onto glass slides. Imaging probes (**e**) without and (**f**) with a microprism mirror attached that allow front- and side-view in the trachea, respectively. (**g**) (*i*) Bright-field and (*ii*) fluorescent images obtained using front-view imaging probe following delivery of Qdot-labeled MSCs into the rat trachea. TR: trachea. (**h**) (*i*) Fluorescent images of 10-μm particles, (*ii*) MSCs, and (*iii*) their seeding density Γ in the rat trachea.

Next, deposition of MSCs onto the rat tracheal surface via liquid delivery was demonstrated. The diameter of the rat trachea (*D* ~ 3.4 mm^39^) is comparable to that of ~5^th^ generation of human lung airway (*D* ~ 3.5 mm^40^). Due to this similarity in size, cell delivery to the rat trachea could be informative for cell seeding in the human small airways. Using a custom-built near-infrared (NIR) imaging system^30^, we visually confirmed that a liquid plug could be effectively formed in the trachea and pushed into downstream airways (Supplementary Fig. 5). Furthermore, we demonstrated liquid plug formation in gelatin tubes with various diameters (3 mm and 6 mm) using a pipette (Supplementary Fig. 6a) or bronchoscope (Supplementary Fig. 6b, Supplementary Video 5), respectively. We used a custom-made imaging setup that employs a Gradient-index (GRIN) lens-based imaging probe (Diameter: ~700 μm) to visualize the cells deposited on the rat trachea^41^. Because of its small diameter, the probe could be easily inserted into the rat trachea (Fig. 5a,b), and was tested for imaging fluorescently labeled 1-μm and 10-μm particles, and Qdot-labeled MSCs in PBS drops placed on a glass slide (Fig. 5c). There was no noticeable difference between the images obtained using the GRIN lens probe and the ones obtained using a conventional fluorescent microscope (Fig. 5d, *i*-*iii*).

The imaging probe (i.e., front-view probe) was modified by attaching a 45°-prism micromirror onto the distal tip for direct perpendicular visualization of the airway surface with reduced optical distortion^42^ (Supplementary Video 6). The modified side-view probe allowed more accurate measurement of the coverage density Γ of cells on the airway^43^ (Fig. 5e,f). The inner surface of the trachea including the cartilaginous rings was visible in a bright-field image acquired using front-view probe (Fig. 5g, *i*, Supplementary Video 7). Qdot-labeled MSCs were administered via plug instillation (plug volume: ~35 μL, instillation flow rate: ~1 mL/s) and allowed to reside onto the trachea for 4–6 hours prior to imaging. The cells were clearly detectable as shown in a fluorescent image (Fig. 5g, *ii*). As the focus of the current study was on the transport behaviors and deposition mechanisms of cells, the longer-term viability and the therapeutic benefits of the delivered cells, which have been extensively shown previously^15,27^, was not assessed in detail.

The intensity of the fluorescent signal obtained was dependent on the radial distance between the imaging probe (working distance: 1 mm) and the surface of the trachea (diameter: ~3.4 mm). Thus, a greater amount of light could be collected from the cells deposited on the airway surface closer to the probe. Meanwhile, because the rat trachea anatomically curves slightly along its axis, it was technically challenging to ensure the exact alignment along the central axis of the trachea while advancing the probe through the trachea. While this optical phenomenon could cause cell deposition appeared to be inhomogeneous on the airway surface, uniform cell seeding across the trachea was visually confirmed from a video obtained (Supplementary Fig. 7, Supplementary Video 8).

Using the side-view probe, we evaluated the surface coverage density Γ of 10-μm particles (concentration: 3.6 × 10^7^ particle/mL) and MSCs (concentration: 2.0 × 10^7^ cell/mL) on the trachea surface following a liquid instillation (Fig. 5h, *i*-*ii*, Supplementary Video 9). For the particles and MSCs, Γ were determined to be approximately 605 particles/mm^2^ and 375 cells/mm^2^, respectively. Greater surface coverage Γ for the 10-μm particles than MSCs could be due to the higher particle concentration used in the experiments (Fig. 5h, *iii*). Because the liquid film thickness^24–26^ and so the seeding density of cells or particles are proportional to the plug capillary number, no discernible inhomogeneity in cell or particle distribution was observed throughout the rat trachea.

### Cell delivery into distal rat lung with transpleural imaging

**Figure 6:**
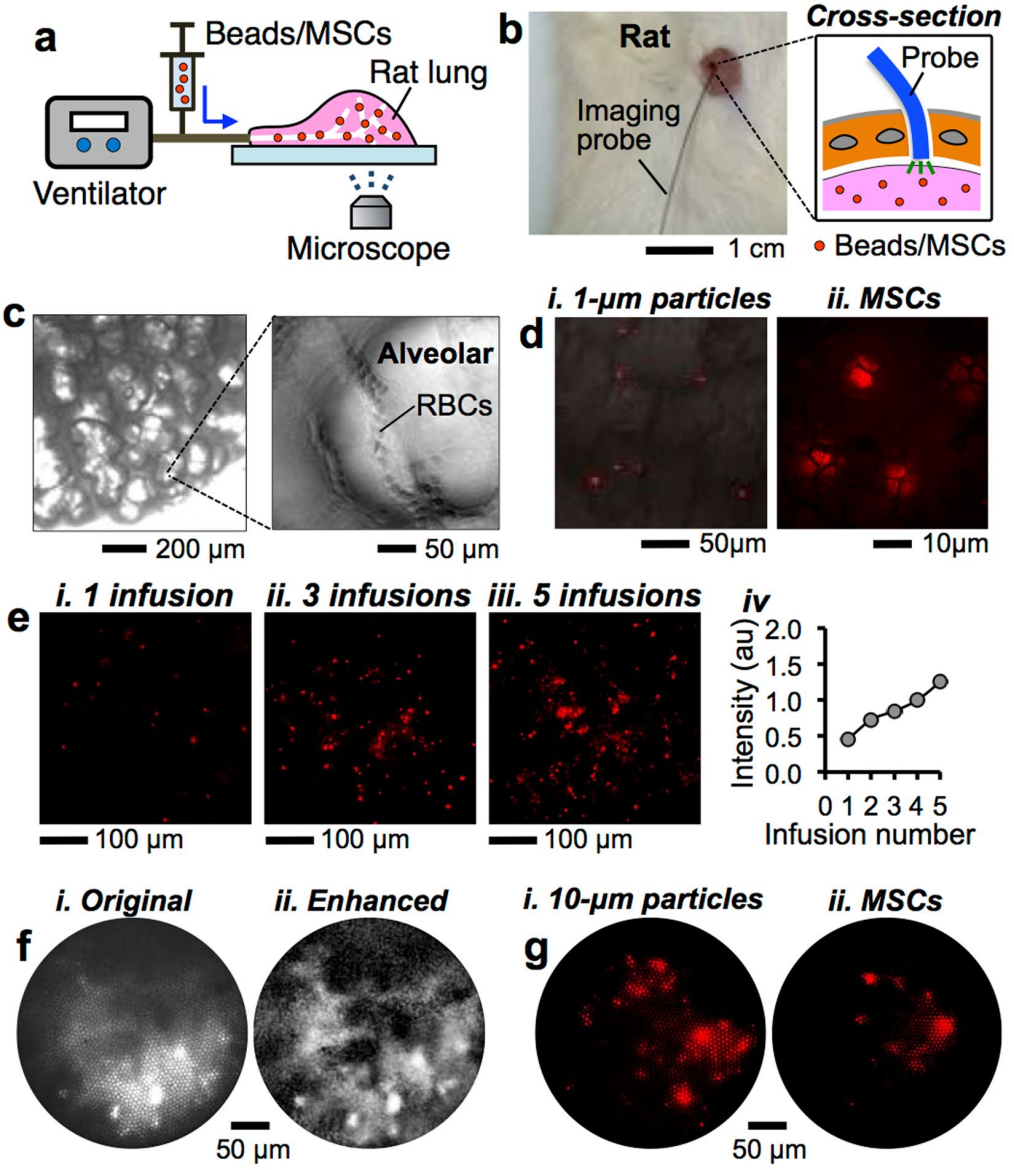
MSC delivery into the rat distal lungs with *in situ* visualization. (**a**) Transpleural imaging of rat alveoli. (**b**) Imaging of rat alveoli using a fiber-optic bundle inserted through a small incision in the chest. (**c**) Bright-field microscopic image of the rat distal lung. Inset: magnified view. (**d**) Microscopic images of rat alveoli deposited with (*i*) 1-μm particles and (*ii*) MSCs. (**e**) Fluorescent images of rat distal lung obtained for different numbers of MSCs that were infused in (*i*) 1 session, (*ii*) 3 sessions, and (*iii*) 5 sessions, and (*iv*) the measured fluorescent signal intensity during infusions. (**f**) Bright-field images of the rat lungs imaged using the fiber-optic imaging bundle (*i*) before and (*ii*) after the image processing. (**g**) Fluorescent images of (*i*) 10-μm particles and (*ii*) MSCs in the distal gas exchange regions (i.e., alveoli).

Following intratracheal delivery of cells into the rat lung, we demonstrated delivery throughout distal gas exchange regions of the lung (i.e., respiratory zone). Approximately 110 μL of cell suspension was instilled at a plug instillation flow rate of ~1 mL/s followed by ~10 min of air ventilation using a previously established protocol^30^. Cells deposited in the distal respiratory zone were visualized by direct extra-pleural imaging (i.e., transpleural imaging)^27^. We employed two different approaches: (*i*) transpleural microscopic imaging (Fig. 6a, Supplemental Fig. 8) and (*ii*) optical fiber based *in situ* transpleural imaging (Fig. 6b). In contrast to the transpleural microscope, optical fiber-based transpleural imaging enabled a minimally invasive approach through a small incision (size: ~1 mm) created in the chest, which was sufficient to insert an optical fiber imaging probe (diameter: ~300 μm). This approach allowed imaging of the distal lung *in situ*.

Because the pleura is very thin (25–85 μm)^44^, the distal lung is optically transparent allowing for visual inspection of alveolar interiors as well as pulmonary capillaries (Fig. 6c). Consequently, intratracheally infused fluorescently labeled 1-μm particles and MSCs deposited within the rat alveoli were clearly visible (Fig. 6d, *i*-*ii*). Using this imaging approach, we confirmed that delivery of the cells could be achieved and that the fluorescent intensity at the distal lung region increased with increased infusion numbers of Qdot-labeled MSCs (plug volume: 120 μL, cell concentration: 2.0 × 10^7^ cell/mL) (Fig. 6e, *i*-*iv*). When smaller plugs (e.g., 35 μL volume) were instilled into the rat lung, no cells were observed in the alveolar regions, while cells were clearly visible in larger airways (e.g., trachea), as shown earlier (Fig. 5g,h). This confirmed that by adjusting the liquid plug volume cells could be delivered into different lung regions selectively (e.g., larger airways vs. alveoli).

Using the optical fiber-based imaging approach, we demonstrated minimally invasive imaging of the distal rat lung. The optical-fiber imaging probe consisted of ~3,000 individual optical fibers (individual fiber diameter: ~5 μm) bundled into a single imaging probe (~300 μm diameter)^45^. Thus, images consisted of honeycomb structures that reduced image quality (Supplementary Fig. 9). To enhance image quality by removing the pixilation artifacts introduced by the honeycomb structure, we employed an image processing technique that is based on a Fourier Transform method^46^ (Supplementary Information). As a result, bright-field images of the distal lung greatly improved such that the air spaces and alveolar boundaries could be clearly identified (Fig. 6f, *i*-*ii*, Supplementary Video 10). In addition, fluorescently labeled 10-μm particles and MSCs administered in the rat alveoli were effectively imaged using this minimally invasive imaging technique (Fig. 6h, *i*-*ii*).

### Cell delivery into human and porcine lungs

**Figure 7:**
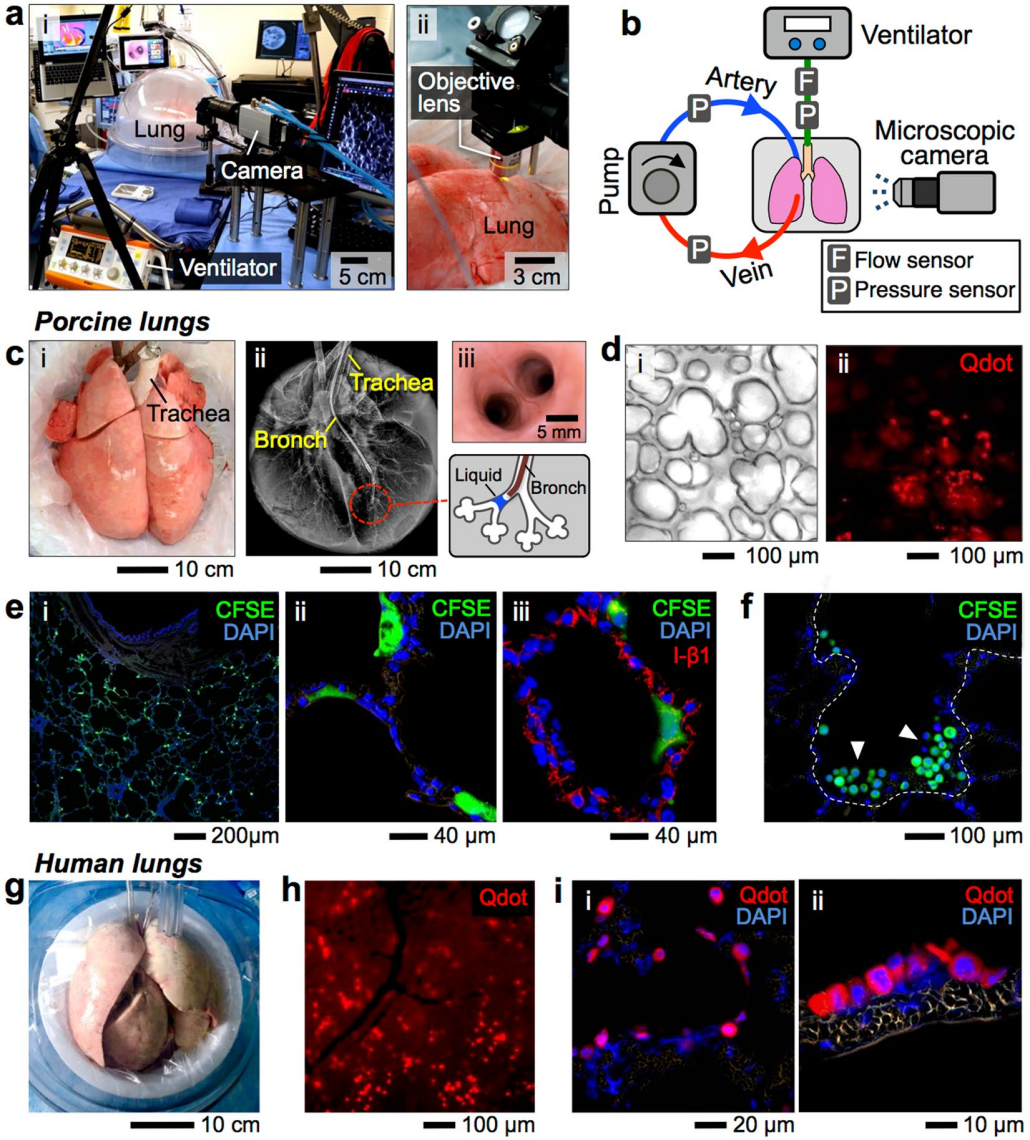
MSC delivery into the porcine and human lungs. (**a**) Images of (*i*) the lung bioreactor used to maintain the viability of the lungs and (*ii*) transpleural cell imaging using our custom-built fluorescent microscope system. (**b**) Experimental setup showing the ventilator, perfusion circuit, and microscopic camera system. (**c**) (*i*) Explanted porcine lung, (*ii*) X-ray image of the lungs with a bronchoscope for cell infusion; inset: schematic of cell injection, and (*iii*) image obtained using the bronchoscope at the cell injection site. (**d**) (*i*) Bright-field and (*ii*) fluorescent images of the porcine lungs. (**e**) Immunostaining images of the lung regions injected with carboxyfluorescein succinimidyl ester (CFSE)-labeled MSCs showing (*i*) uniform distribution of the cells throughout the lung, (*ii, iii*) adhesion of the cells on the alveolar surfaces. (**f**) Aggregation of the cells by gravity settling. (**g**) Explanted human lungs prepared for the experiment. (**h**) A fluorescent image showing Qdot-labeled MSCs delivered into the alveoli. (**i**) (*i*-*ii*) Immunostaining images of the lung regions injected with the Qdot-labeled MSCs.

To demonstrate the utility of our methods in a clinically relevant model, we utilized human lungs rejected for transplant and healthy porcine lungs *ex vivo*. In both cases, lungs were harvested in a standard clinical fashion, under institutionally approved protocols^47,48^. Fluorescently labeled MSCs were delivered via intratracheal liquid instillation and subsequently imaged using the minimally invasive transpleural imaging approach developed in this study. To maintain the viability of the lungs during experiments, explanted lungs were supported using an *ex vivo* lung perfusion (EVLP) platform developed by our research group^14,49,50^ (Fig. 7a, *i*-*ii*, Supplementary Video 11). While on EVLP, the trachea is connected to a ventilator that supplies oxygen to the lungs at a standard respiratory rate (8–12 bpm) and tidal volume (6 mL/kg). The lungs were maintained with a positive end-expiratory pressure (PEEP) of 5 cmH_2_O, which is the standard setting for a positive pressure ventilator in critical care, enabling ventilation of the whole lung and precluding any atelectatic regions. To preserve the lung structure and maintain tissue viability, the lung vasculature was continuously perfused with Perfadex (human lungs) or porcine blood (porcine lungs) via a pump connected to the cannulae placed within the pulmonary artery and veins. Following intratracheal administration of MSCs, a custom-built fluorescent microscopic camera system was used to verify cell delivery into the distal gas exchange regions of the lungs (Fig. 7b).

Using a flexible video bronchoscope (diameter: 3.8 mm), we intratracheally instilled MSCs (volume: 120 μL, concentration: 2.0 × 10^7^ cell/mL) into explanted porcine lungs (Fig. 7c, *i*). To verify the cell injection sites in the lung prior to delivery, X-ray images were obtained confirming the location of the bronchoscope within targeted lung regions (Fig. 7c, *ii*). As shown in the X-ray image, the bronchoscope is small enough to reach distal regions in every segment of human and porcine lung, and therefore has utility for liquid instillation. A liquid plug can be injected into smaller airways using a narrow tube (diameter: <1.8 mm), which can be inserted through the working port (diameter: 1.8 mm) of the bronchoscope. Our research team accomplishes plug injection using this bronchoscope both in the research and clinical settings^14^. In addition, smaller bronchoscopes (e.g., device diameter: 3.1 mm, working channel diameter: 1.2 mm) could be used to improve access to even smaller airways in the human lungs^51^. Video bronchoscopy enabled *in situ* visualization of cell injection via liquid instillation (Fig. 7c, *iii*, Supplementary Fig. 10). Because the pleura is highly transparent, as shown in the bright-field images of the lung (Fig. 7d, *i*), Qdot- labeled MSCs injected into the alveoli were also clearly visible by our custom-built imaging system, confirming the effectiveness of cell delivery into selected lung regions using the small-volume liquid instillation approach (Fig. 7d, *ii*). Following cell injection, lungs were maintained on EVLP for 4–6 h to allow cell attachment onto the surfaces of the airways or alveoli. Histologic analysis was conducted in the lung regions injected with MSCs labeled with carboxyfluorescein succinimidyl ester (CFSE), which is a commercially available membrane-permeable fluorescent cell labeling dye. CFSE is taken up by live cells, cleaved by intracellular esterases to become membrane impermeable, and thus retained as a long-term fluorescent label within live cells without being transferred to adjacent cells (Supplementary Fig. 11).

As shown by fluorescent microscopy, CFSE-labeled MSCs were distributed uniformly throughout the distal lung (Fig. 7e, *i*-*ii*). The flattened cell morphology and co-localization of MSCs with integrin beta 1 (I-β1), which is a known mediator of MSC migration through parenchymal tissues^52^, suggest that cells administered were adherent to alveolar surfaces (Fig. 7e, *iii*). Magnified immunofluorescent micrographs show clearly distinct overlapping foci (‘co-localization’) of green (fluorescently-labeled MSC) and red (I-β1) staining – resulting in yellow-orange punctate signal (Supplementary Fig. 12). Although I-β1 was not expressed exclusively by MSCs, this suggests that MSCs co-localized with I-β1. While cells were distributed relatively uniformly throughout the distal lung via liquid plug instillation, injected cells could accumulate non-uniformly on the side of the airways at certain local regions (Fig. 7f). This cell accumulation with a directional bias could be due to gravity settling as discussed (Figs 3 and 4) or asymmetric splitting of the liquid plug at the airway bifurcation due to different angles relative to the direction of gravity^26^. We also infused MSCs into explanted human lungs and visually confirmed uniform cell delivery (Fig. 7g). Qdot-labeled MSCs were clearly seen through the transparent pleura of the human lung, confirming the delivery of cells into selected lung regions in real time (Fig. 7h). In addition, immunostaining of human lung samples from cell delivery sites demonstrated cells attachment to the airway surfaces (Fig. 7I, *i*-*ii*).

## Discussion

In this study, we provide theoretical and experimental investigations into the transport behaviors of cells introduced through the lung airways. This report also presents the development of a small scale, real-time, minimally invasive imaging modality that could enable long-term monitoring of cells administered into the lung (Fig. 1). We used a custom-built imaging system incorporating LED and laser sheet imaging modalities to investigate cell transport and deposition (Fig. 2). We showed that the cell seeding density was proportional to the plug instillation speed and cell concentration (Figs 3 and 4). Using small and large animal lungs, we demonstrated cell delivery and visualization *in situ*. We first demonstrated delivery of Qdot-labeled MSCs into the rat trachea (Fig. 5) and distal alveoli (Fig. 6). Cells deposition on the surfaces of the trachea and alveoli was confirmed using optical fiber-based imaging probes and transpleural imaging techniques. Finally, using explanted porcine and human lungs we demonstrated that cell delivery via liquid instillation and minimally invasive imaging techniques could be employed in clinically relevant models (Fig. 7).

Given the therapeutic potential of cell-based therapy for treating lung disease, there is a need to further characterize cell seeding behavior and dosing effects. To the best of our knowledge, this is the first study providing direct insights into the cell transport and deposition mechanics during cell delivery through the pulmonary airways. Our study was motivated by (*i*) the limited therapeutic efficacies of MSCs thus far reported in clinical studies and (*ii*) experimental evidence suggesting that therapeutic efficacy may be influenced by cell dosage and delivery technique. Our theoretical and experimental results demonstrate that the cell dose can be effectively controlled by the instillation flow condition and the cell concentration in suspension. In addition, cell delivery into selected lung regions could be achieved by selecting the liquid volume and instillation speed based on the theoretical model, and targeted delivery was confirmed by using our minimally invasive imaging modalities.

While cell seeding can be effectively achieved in the lung via liquid plug instillation, this method would be most suitable for smaller airways. For larger airways (e.g., human trachea or intralobar bronchi), where formation of a liquid plug can be challenging, fluid with high viscosity and surface tension could be used to facilitate plug generation^53^. Alternatively, cell delivery and retention in larger airways could be achieved by draining a fluidic cell carrier, where liquid motion induced by gravity could result in cell delivery via film deposition without requiring plug formation^54^. In this current study, however, we focused on elucidating transport behaviors and deposition mechanisms of intratracheal cell delivery into the distal lung, which accounts for the vast majority of treatable lung volume. Accordingly, gelatin tubes (e.g., 1-mm tube) used in this study served as tubular structures representative in scale of human small airways, thus enabling us to investigate cell transport and deposition in the regime of conducting airways most abundant throughout the lung.

With enhanced understanding of cell delivery mechanisms, liquid plug delivery allows for (*i*) delivery of therapeutic cells throughout all lung regions in a dose- and region-specific manner and (*ii*) delivery of microliter volumes to avoid deleterious effects of large volume liquid instillation on gas exchange and lung compliance. Minimally invasive imaging techniques developed and implemented in this study would enable visual inspection of the lung for extended periods of time, allowing for the real-time monitoring of cell migration, engraftment, and therapeutic action (e.g., using fluorescent probes to detect protein or gene activation) in real time. Although therapeutic effects of MSCs administered into the lungs using our approach would need to be investigated thoroughly, we envision that our findings could enhance the clinical utility of stem cell-based therapies and aid in lung bioengineering, and ultimately contribute to efforts to reduce the global burden of respiratory diseases.

## Methods

### Custom-built fluorescent imaging system

The imaging system was constructed using commercially available optical parts and related hardware. The laser light sheet was created by passing the beam of a laser (Jive 561 nm 200 mW laser, Cobolt) through a cylindrical lens (ACY254-050-A, Thorlabs). For epi-illumination, a LED (M565L3, Thorlabs) was used as a light source. Fluorescent signals from fluorescent microbeads (Excitation/Emission: 580 nm/605 nm) with diameters of 1 μm (F8821, Thermo Fisher Scientific) and 10 μm (F8834, Thermo Fisher Scientific), and MSCs labeled with 655-nm Qdots (Q21321MP, Thermo Fisher Scientific) were separated from the excitation light by using a dichroic mirror (FF596-Di01-25 × 36, Semrock). The separated emission light signals were passed through an optical filter (FF02-641/75-25, Semrock) and detected by a camera (Zyla sCMOS 4.2, Andor) with the10× (PlanN 10×, NA 0.25, Olympus) and 20× (LUCPlanFLN 20×, NA 0.45, Olympus) objectives.

### Fiber-optic imaging setup

For minimally invasive imaging, a GRIN lens (LRL-070-P300, GoFoton) and optical-fiber imaging bundle (FIGH-03-215S, Fujikura) were used as imaging probes. These imaging probes were integrated into the epi-illumination compartment of the custom-built fluorescent imaging microscope, where the distal end of the probe (i.e., imaging tip) is inserted into the animal and images formed at the proximal end of the probe are detected by using the objective lenses and camera. The side-view imaging probe was constructed by gluing a right angle micro-prism mirror (8531-601-1, Precision Optics) coated with aluminum onto the distal imaging tip of the front-view imaging probe. The attachment of the mirror and imaging probes were achieved via optical adhesive (NOA 68, Norland) that was cured by exposure to UV light using a hand-held UV gun (86–884, Edmund Optics).

### Gelatin channel fabrication

Gelatin channels were fabricated using the molding technique. A mold was prepared using a silicon container and a cylindrical plastic tube (outer diameter: 1 mm) in which 10 wt% molten gelatin in PBS was crosslinked into a tubular channel structure. To prepare 10 wt% gelatin solution, 10 g of gelatin powder (G2500, Sigma-Aldrich) was dissolved in 100 mL of PBS buffer heated to ~60 °C. Then, the gelatin solution was poured into the mold and allowed for crosslinking at 4 °C for ~30 min. After crosslinking, the plastic tube was gently slipped out and the gelatin channel block was removed from the mold. The gelatin block was then placed in a Petri dish at room temperature (~24 °C) for ~15 min before one end was connected to a syringe pump (GenieTouch Syringe Pump, Kent Scientific) via a polymer tubing (Masterflex, Cole-Parmer) for *in vitro* cell delivery experiments.

### MSC delivery and labeling

MSCs derived from rat bone marrow (RASMX-01101, Cyagene Biosciences), porcine adipose tissue, or human adipose tissue, were expanded up to passage 3 before use. At the time of the experiment, cells were trypsinized, counted with the Countess™ Automated Cell Counter (Invitrogen), and suspended in PBS at a final concentration of 2.0 × 10^7^ cells/mL for labeling with carboxyfluorescein succinimidyl ester (CSFE) (ab113853, Abcam) or carboxyl Qdot solution (Q21321MP, Invitrogen) according to the manufacturers’ instructions. Cell delivery in gelatin channels and rodent lungs was accomplished utilizing a 1-mL syringe. In porcine and human lungs, a 3.8 mm flexible bronchoscope with a 1.2 mm lumen for therapeutic delivery and intervention was used (Ambu® aScope™3, Ambu).

### Rat lung harvest

Lungs were harvested from 12-week old Sprague-Dawley rats weighing 250–350 g (Charles River). Animals were euthanized via intraperitoneal injection of sodium pentobarbital (Sigma, 140 mg/kg) after heparin injection (1000 U/kg, Sagent Pharmaceuticals). Immediately after euthanasia, a median sternotomy was performed, and the trachea was exposed and cannulated using a 16–18 g catheter. Ventilation was performed using a small animal ventilator (Harvard Inspira ASV, Harvard Apparatus). All animal experimental work was conducted under protocols approved by the Columbia University Institutional Animal Care and Use Committee (IACUC) and compliant with the NRC *Guide for the Care and Use of Laboratory Animals*, eighth edition. All methods were performed in accordance with the relevant guidelines and regulations.

### Porcine lung harvest

Yorkshire pigs (5–7 months of age, weighing 40–60 kg) underwent general anesthesia via intramuscular induction with Telazol (5 mg/kg, Zoetis) and buprenorphine hydrochloride (0.03 mg/kg, Hospira), and maintenance with continuous intravenous infusions of fentanyl citrate (0.1 mg/kg/h, West-Ward,), midazolam (1.5 mg/kg/h, Akorn), and inhaled isoflurane (1–5% in oxygen, Henry Schein). A median sternotomy was performed. A bolus of heparin (30,000 U IV) was given (Sagent Pharmaceuticals), and a cannula was placed and secured within the main pulmonary artery. Blood was collected in citrate phosphate dextrose (CPD) collection bags (Chinook Medical) and stored at 8 °C. Once a non-perfusing rhythm was observed, a cold anterograde low-potassium dextran flush (Perfadex; Vitrolife) with alprostadil (25 mg/kg, Prostin VR Pediatric^®^, Pfizer) was given, and the appendage of the left atrium was cut. Topical cooling was applied, the lungs were inflated to a sustained airway pressure of 15 cmH_2_O, and the trachea was stapled (Endo GIA™ device, Medtronic). The heart and lungs were explanted en bloc and placed on ice on a sterile table. The heart was removed and arterial and venous cannulas were secured. Lungs were then placed on the *ex-vivo* lung perfusion (EVLP) system and perfused with whole blood collected at the time of donor lung harvest.

### Human lung harvest

Human lungs rejected for transplantation on the basis of standard clinical criteria were procured using standard protocols. The lungs were prepared and cannulated in a similar method as previously described^14,49^ and placed on an EVLP system to be ventilated and perfused with Perfadex. The collection and use of human tissue for research received approval from the Institutional Review Board (IRB) at Columbia University and LiveOnNY organ donor network. Informed consent was obtained from all donors or authorized representatives of the donors.

### Immunohistology

Lung sections were de-paraffinized, subjected to boiling citrate buffer (pH = 6.0) for antigen retrieval, and blocked with 10% normal goat serum in PBS for 2 hr at room temperature. The primary antibody, mouse anti-integrin β−1/CD29 (MAB17783), was added and incubated for 12 h at 4 °C, or 4 hr at room temperature at a 1:100 dilution. For all stains, the secondary antibody, goat anti-mouse IgG 647 (ab150116), was diluted 1:200 and incubated for 1 hr at room temperature. Sections were mounted in Vectashield Mounting Medium with DAPI (Vector Laboratories), coverslipped, and imaged using an Olympus FSX100 or BX61VS microscope (Olympus).

### Particle and cell settlement simulation

MATLAB software (MathWorks) was used to simulate the settlement of microparticles and MSC. The MATLAB code used for the simulation is provided in the Supplementary Information.

### Image processing for enhancing image contrast

To enhance the quality of the images obtained using a fiber-optical imaging bundle, a Fourier Transform method was employed that can effectively remove pixilation artifacts in the images. The image processing was conducted using a code written in Python programming language (please see Supplementary Information).

## Electronic supplementary material

Supplemental figures, computer codes and captions for videos

Video 1

Video 2

Video 3

Video 4

Video 5

Video 6

Video 7

Video 8

Video 9

Video 10

Video 11
