# Supplementary material for: Controlled delivery and minimally invasive imaging of stem cells in the lung
Source: Sci Rep. 2017 Oct 12;7:13082. doi: 10.1038/s41598-017-13280-9 (PMC5638808; doi:10.1038/s41598-017-13280-9)
Supplement: Supplementary file 1 — Supplemental figures, computer codes and captions for videos [file 41598_2017_13280_MOESM1_ESM.pdf]

Controlled delivery and minimally invasive imaging of stem cells in the lung

Jinho Kim[1], Brandon Guenthart[2], John D. O'Neill[1], N. Valerio Dorrello[1,3], Matthew Bacchetta[2], and Gordana Vunjak-Novakovic[1,4,*]

Departments of [1]Biomedical Engineering, [2]Surgery, [3]Pediatrics, and [4]Medicine, Columbia University, New York, NY, USA
*Corresponding author: gv2131@columbia.edu

Supplementary information

Supplementary Figures

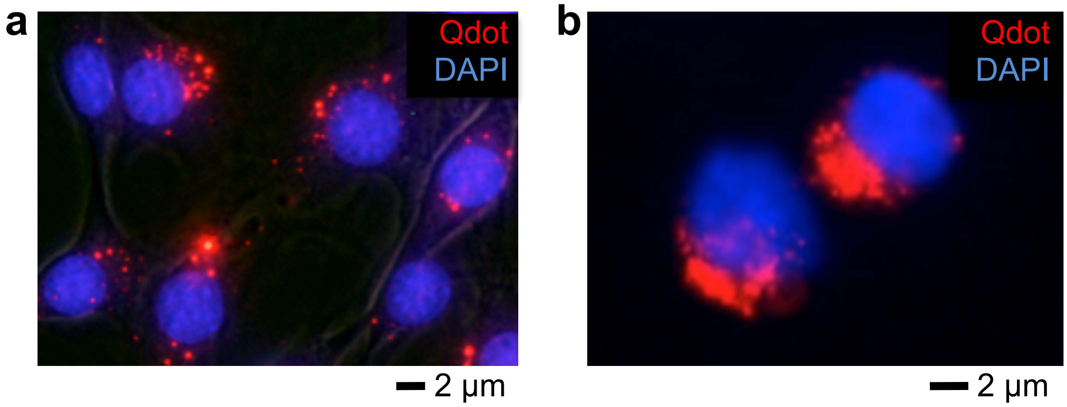

Supplementary Figure 1. Qdot-labeled MSCs stained with DAPI (**a**) attached to a Petri dish and (**b**) suspended in cell culture media.

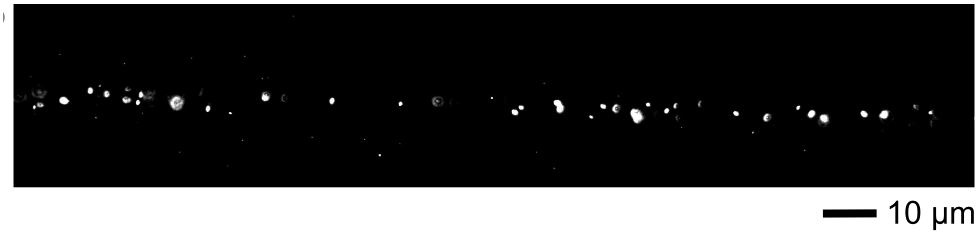

Supplementary Figure 2. Characterization of the laser-sheet imaging method. Thickness of the laser-light sheet was measured to be ~5 μm by illuminating the light sheet to a gelatin tube filled with 1- μm particles suspended in a liquid plug.

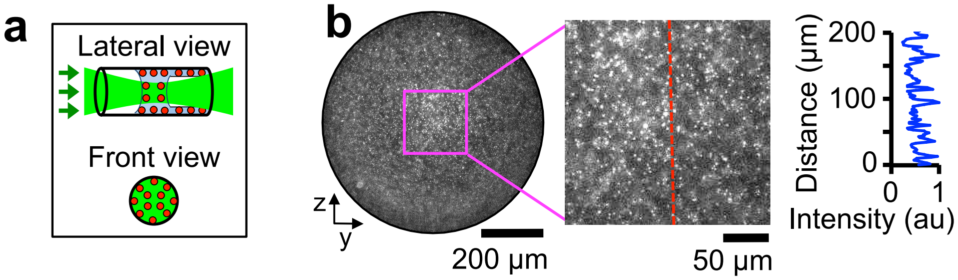

Supplementary Figure 3. Characterization of the LED imaging method. (**a**) Light path of the LED imaging for visualizing a liquid plug within a gelatin channel and (**b**) 1-μm particles suspended in a liquid plug within the channel imaged. Signal intensity along the vertical centerline (red doted line) is plotted.

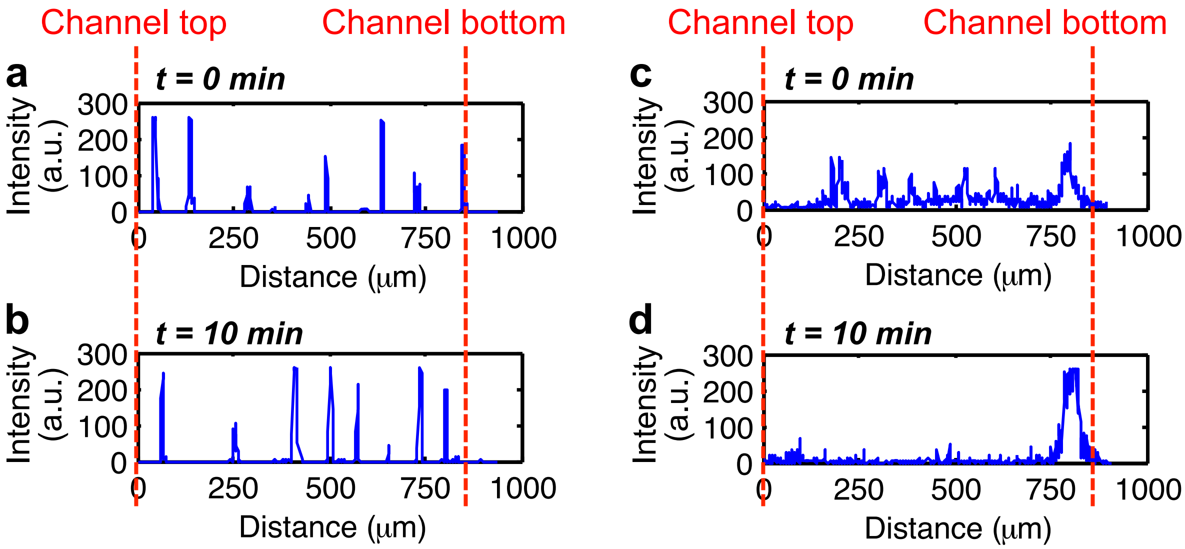

Supplementary Figure 4. Distribution of microparticles and MSCs by gravity across the channel. Fluorescence intensity profiles of 1-μm particles at (**a**) *t* = 0 min and (**b**) *t* = 10 min, and MSCs at (**c**) *t* = 0 min and (**d**) *t* = 10 min within the channel.

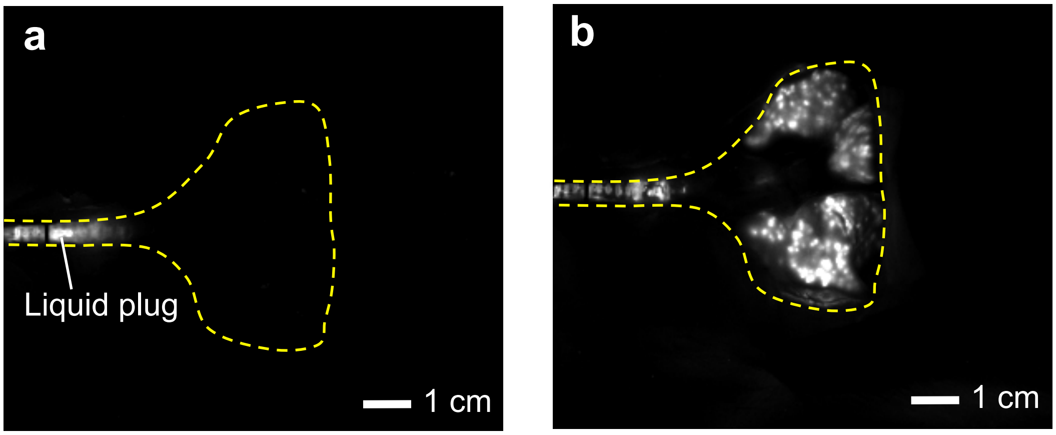

Supplementary Figure 5. Liquid delivery into the rat lung by instillation of a liquid plug generated in the trachea. Our custom-built near-infrared imaging system was used to visualize (**a**) a liquid plug formed in the rat trachea and (**b**) liquid film generated in the lung following instillation via airflow.

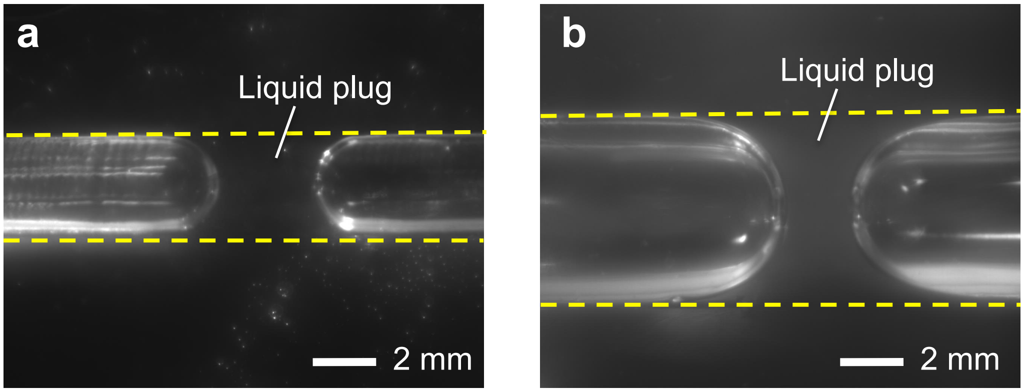

Supplementary Figure 6. Liquid plug formation in gelatin channels. Liquid plugs (volume: ~ 40 μL) were formed in gelatin channels with diameters of (**a**) 3 mm and (**b**) 6 mm using a micropipette and a bronchoscope, respectively.

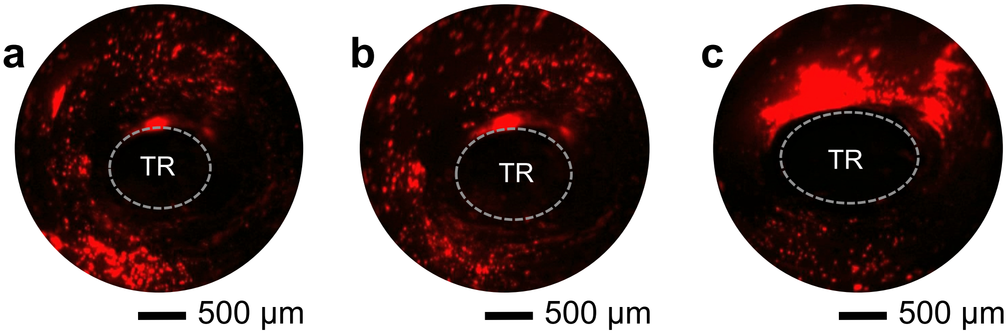

Supplementary Figure 7. Images of Qdot-labeled MSCs deposited in the trachea. (a-c) Fluorescent images were obtained by translating the front-view imaging probe through the rat trachea following instillation of Qdot-labeled MSCs.

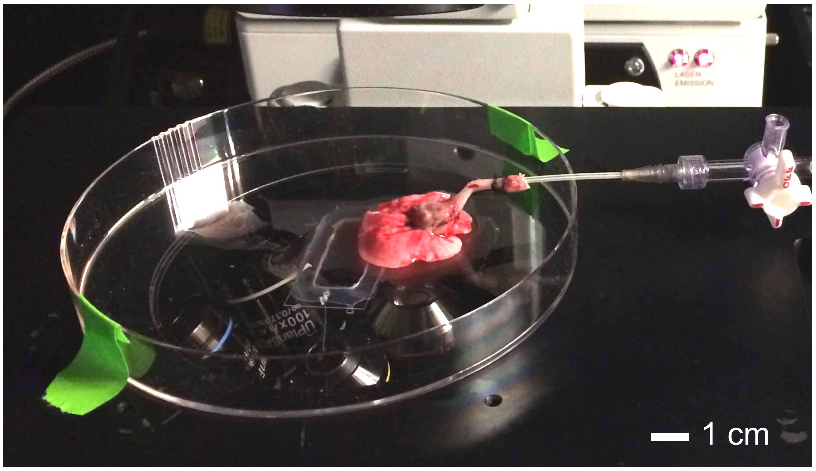

Supplementary Figure 8. Explanted rat lungs placed on a Petri dish for transpleural imaging.

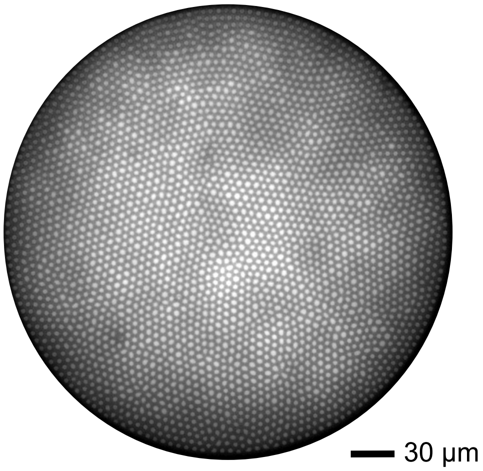

Supplementary Figure 9. The honey-comb structures between the individual fibers are shown in an image obtained using the optical-fiber imaging probe.

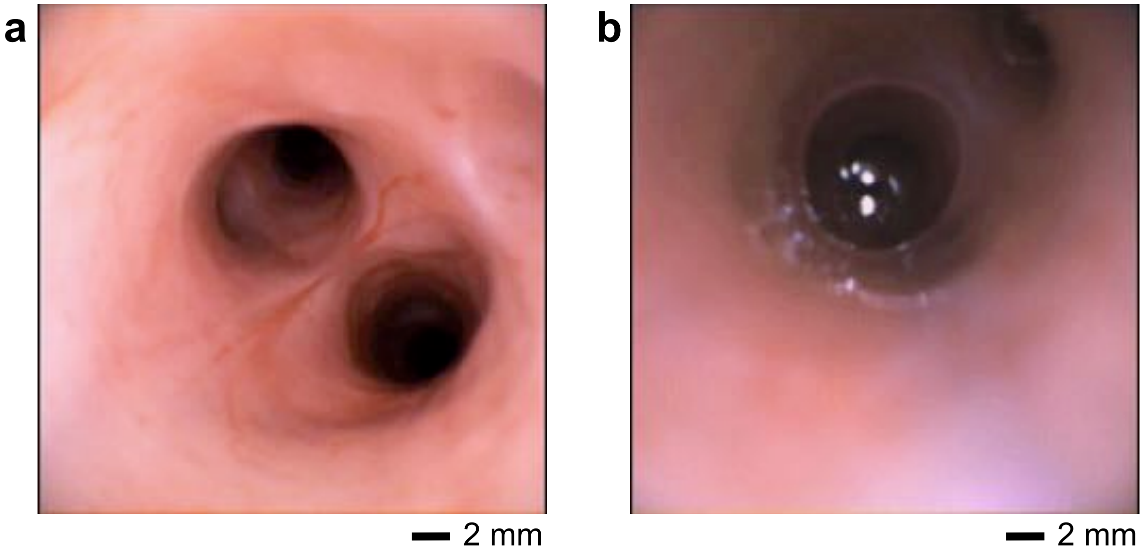

Supplementary Figure 10. Images of porcine lung airways obtained using a bronchoscope. (**a**) An image of the airways where the bronchoscope was placed for cell instillation. (**b**) Image of a small-volume of liquid carrying cells and being instilled from the distal tip of the bronchoscope. The white light was reflected on the meniscus of the liquid plug.

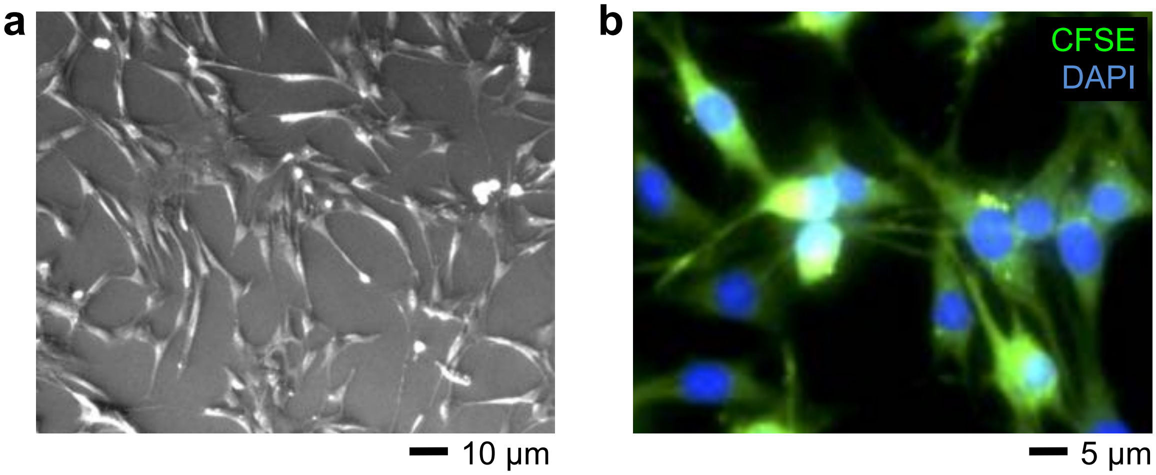

Supplementary Figure 11. CFSE-labeled MSCs. (**a**) A bright-field and (**b**) fluorescent microscopic images of MSCs labeled with CFSE.

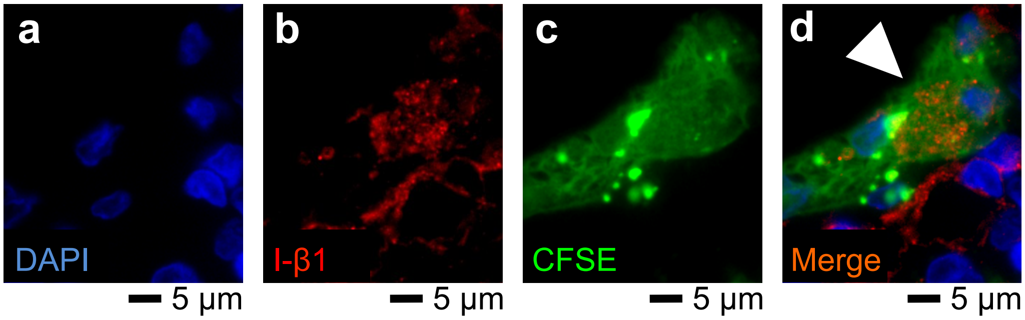

Supplementary Figure 12. Immunostaining images of a MSC administered into the lung. (**a**) DAPI, (**b**) integrin-β1 (I- β1), (**c**) CFSE, and (**d**) merged images. A white arrowhead indicates the region with visible co-staining of I-β1 and MSC.

Supplementary Materials

MATLAB code used for particle and cell settlement simulation

```
tau = 1;                                   % time interval in seconds
N=15;                                      % number of time steps
tf=tau*N;                                  % total time
time = tau * 1:N;                          % time vector for plotting
dimensions = 2;                            % two dimensional simulation
g=9.81;                                    % gravity
rhop=1012; rhof=1000; drho=rhop-rhof;      % effective density of a particle or cell
d=10.0e-6;                                 % particle diameter in meters
eta=9e-4;                                  % viscosity of water at 25°C
kB=1.38e-23;                               % Boltzmann constant
T=300;                                     % Temperature in degrees Kelvin
D=kB*T/(3*pi*eta*d);                       % Diffusivity of a particle
LD= sqrt(D*dimensions*tau);                % diffusion length
Vsett = (drho*g*d^2)/(18*eta);             % particle settling velocity
u0=200*10^-6;                              % air flow velocity
Trelax = (drho*d^2)/(18*eta);              % particle relaxation time
dx = LD*(rand(N,1)*2-1)+u0*tau;
dy = LD*(rand(N,1)*2-1)-(Vsett+Trelax*u0)*tau;
x = cumsum(dx);
y = cumsum(dy);

Animation generation
axis manual
axis equal
hold on
axis([-100*Dav 4000*Dav -200*Dav 200*Dav])

for i = 1:1:N
hm=plot(x(i),y(i),'-o','LineWidth',2,'MarkerEdgeColor','k','MarkerFaceColor','r','MarkerSize',8);
pause(0.1)
grid on
grid minor
end
```

Fourier Transform method for removal of pixilation artifacts in images

To eliminate the pixelation artifacts introduced by the honeycomb structures, we first converted the RGB spectrum of the image obtained using the optical fiber imaging probe into grayscale. This image conversion enabled a Discrete Fourier Transform in 2D on the original images using the following equation:

$$F(u,v) = \iint_{-\infty}^{\infty} f(x,y)e^{-j2\pi(ux+vy)}dxdy$$

where *u* and *v* are spatial frequencies in x and y directions, respectively. The transformed images are reoriented to place the zero frequency components of the matrix of pixels in the center of the new image matrix. Taking the natural logarithm of the absolute value of the matrix, we can then obtain a magnitude spectrum of the original image as shown below.

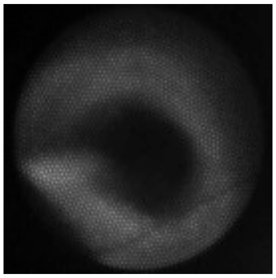
Original image

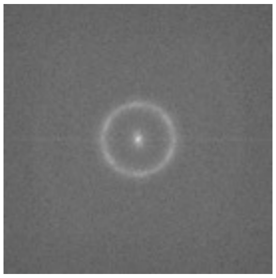
Magnitude spectrum

In the magnitude spectrum above, the center point represents the desired image while the surrounding outer circle represents the unwanted honeycomb pixelation frequency. To remove the unwanted frequencies, we filtered out the outer circle from the magnitude spectrum (below). Following the filtering, we reconstructed the image using an inverse Fourier Transform using the equation;

$$f(x,y) = \iint_{-\infty}^{\infty} F(u,v)e^{-j2\pi(xu+yv)}dudv$$

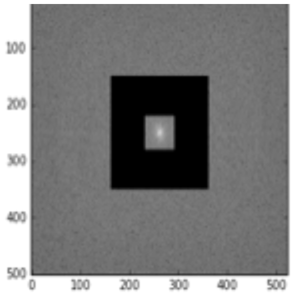

Filtered magnitude spectrum

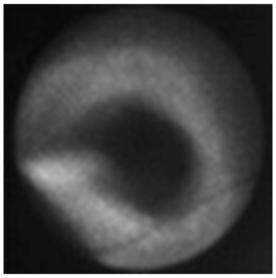
Reconstructed image

Alternatively, we created a refined band-pass filter by selecting a threshold value that is then multiplied by the mean pixel values of the image. We then iterated through the pixels of the magnitude spectrum, selecting only pixels above the threshold, thus creating a matrix of boolean values to display only the bright elements of the original image. To preserve desired features in the image, we set the center point values in the boolean matrix to “False” and then applied the filter to the Fourier shifted image, setting all “True” values to “Zero”. Finally, the image was reconstructed using an inverse Fourier Transform.

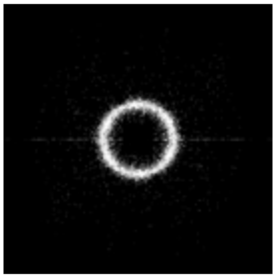

Threshold magnitude spectrum

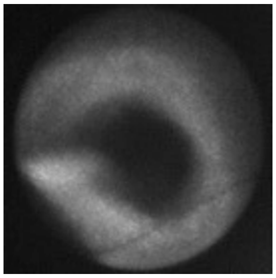

Reconstructed image

Python code used for the image processing

```
from matplotlib import pyplot as plt
import matplotlib.pyplot as plt
import matplotlib.image as mpimg
import numpy as np
import scipy.misc
from scipy import ndimage

def rgb2gray(rgb):
   return np.dot(rgb[...,:3], [0.299, 0.587, 0.114])

def fourier(image):
        img = mpimg.imread(image, 0)
        imgray = rgb2gray(img)
        f = np.fft.fft2(imgray)
        return f

def fouriershift(image):
        img = mpimg.imread(image, 0)
        imgray = rgb2gray(img)
        f = np.fft.fft2(imgray)
        f2 = np.fft.fftshift(f)
        return f2

def magspec(image):
        mag = np.log(np.abs(fouriershift(image)))
        return mag

def halfrows(image):
        f = fourier(image)
        r = f.shape[0]/2      # number of rows/2
        return r

def halfcols(image):
        f = fourier(image)
        c = f.shape[1]/2      # number of columns/2
        return c

#globals
n = 30
thresh = 1.15

def showfilter(image):
```

```
        r = halfrows(image)
        c = halfcols(image)
        filt = magspec(image) > thresh*(np.mean(magspec(image)))
        filt[(r-n):(r+n), (c-n):(c+n)] = False
        return filt

def bandpass(image):
        mag = magspec(image)
        r = halfrows(image)
        c = halfcols(image)
        f2 = fouriershift(image)
        filt = mag > thresh*(np.mean(mag)) #taking all points above threshold
        filt[(r-n):(r+n), (c-n):(c+n)] = False #preserving centerpoint/desired image
        f2[filt] = 0 #eliminating outer ring aka honeycombs
        invshift = np.fft.ifftshift(f2)
        finimg = np.real(np.fft.ifft2(invshift))
        return finimg

def saveimgmag(image, name):
        scipy.misc.imsave(('testimgs/final/' + name + 'mag.jpg'), magspec(image))

def savefilteredimg(image, name):
        scipy.misc.imsave(('testimgs/final/' + name + '.jpg'), bandpass(image))

def savefilter(image, name):
        scipy.misc.imsave(('testimgs/final/' + name + 'filter.jpg'), showfilter(image))

savefilter('imgs/trachea.jpeg', 'trachea')
saveimgmag('imgs/trachea.jpeg', 'trachea')
savefilteredimg('imgs/trachea.jpeg', 'trachea')
```

Supplementary Video Legends

Supplementary Video 1. Deposition of 1-μm particles onto the surface of a gelatin tube (diameter: 1 mm) via liquid plug instillation.

Supplementary Video 2. 1-μm particle suspended in a liquid plug.

Supplementary Video 3. 10-μm particle suspended in a liquid plug.

Supplementary Video 4. A MSC suspended in a liquid plug.

Supplementary Video 5. Liquid plug formation in a gelatin tube (diameter: 6 mm) using a video bronchoscope (diameter: 3.8 mm).

Supplementary Video 6. Assembly of the side-view imaging probe.

Supplementary Video 7. A bright-field video obtained within the rat trachea using a front-view imaging probe.

Supplementary Video 8. A fluorescent video obtained within the rat trachea using a front-view imaging probe after seeding of Qdot-labeled MSCs.

Supplementary Video 9. A fluorescent video obtained within the rat trachea using a side-view imaging probe after seeding of Qdot-labeled MSCs.

Supplementary Video 10. A bright-field video obtained at the distal lungs of the rat lung using the optical-fiber imaging probe.

Supplementary Video 11. The porcine lungs supported on EVLP.
